# Influence of surfactants addition on the properties of calcium hypochlorite solutions

**DOI:** 10.1590/1678-7757-2018-0157

**Published:** 2019-01-07

**Authors:** Júlia Eick Iglesias, Lucas Siqueira Pinheiro, Daniel Eduardo Weibel, Francisco Montagner, Fabiana Soares Grecca

**Affiliations:** 1 Universidade Federal do Rio Grande do Sul, Faculdade de Odontologia, Departamento de Odontologia Conservadora, Porto Alegre, Rio Grande do Sul, Brasil.; 2 Universidade Federal do Rio Grande do Sul, Instituto de Química, Departamento de Físico-Química, Porto Alegre, Rio Grande do Sul, Brasil.

**Keywords:** Calcium hypochlorite, Endodontics, Root canal irrigants, Sodium hypochlorite, Surface tension

## Abstract

**Objectives:**

The aim of this study was to evaluate the influence of surfactants 0.2% or 0.1% cetrimide (Cet) or 0.008% benzalkonium chloride (BAK) on 2.5% calcium hypochlorite (Ca(OCl)_2_), and compare to sodium hypochlorite (NaOCl), regarding the properties of pH, free chlorine content, surface tension, contact angle, pulp dissolution and antimicrobial activity.

**Material and Methods:**

The pH and free chlorine content were evaluated by digital pHmeter and by titration, respectively. Surface tension was measured by the platinum ring technique with a Du Noüy tensiometer. The solution's contact angle in human dentin surfaces was checked by Drop Shape Analyzer software. Bovine pulps were used for pulp dissolution analysis and the dissolving capacity was expressed by percent weight loss. Antimicrobial activity over *Enterococcus faecalis* was evaluated by the agar diffusion method.

**Results:**

Surfactants addition to Ca(OCl)_2_ and NaOCl did not alter the pH, free chlorine content and pulp dissolution properties. Ca(OCl)_2_ had the highest surface tension among all tested solutions. When surfactants were added to Ca(OCl)_2_ and NaOCl, there was a significant reduction of surface tension and contact angle values. The addition of 0.2% or 0.1% Cet enhanced antimicrobial activity of both Ca(OCl)^2^ and NaOCl.

**Conclusion:**

Surfactant addition to 2.5% Ca(OCl)^2^ has shown acceptable outcomes for pH, free chlorine content, surface tension, contact angle, pulp dissolution and antimicrobial activity. Furthermore, the addition of 0.2% Cet showed better results for all tested properties.

## Introduction

Root canals present a complex internal anatomy with irregularities and isthmus, which may not be accessible during instrumentation. Hence, irrigation is an important step of the root canal treatment and should allow the irrigant to reach those areas.^1^ The ideal root canal irrigant should have antimicrobial spectrum, including action against biofilms, dissolve pulp and necrotic tissue, inactivate endotoxin, prevent/remove smear layer and not injure the periapical tissue in case of accidental contact.^1^ Moreover, irrigants with lower surface tension may penetrate more in dental tubules, with greater antimicrobial activity.^2^

Sodium hypochlorite (NaOCl) is the most used irrigant solution in clinical practice, because it has the unique capacity to dissolve necrotic tissue and organic components of the smear layer.^1^ However, its chemical instability can influence the availability of chlorine ions and interfere with the desired characteristics of the irrigant.^3^ In this regard, other auxiliary chemicals should be studied.

Calcium hypochlorite (Ca(OCl)_2_) is a white powder that can be dissolved in distilled water, used for industrial sterilization and purifying water treatment.^4^ It is relatively stable and shows more available chlorine content than NaOCl.^3^ ^,^ ^5^ Studies demonstrated the potential of Ca(OCl)_2_ solutions to dissolve tissue^5^ ^,^ ^6^ and antimicrobial action against *E. faecalis* ^7^. Also, Ca(OCl)_2_ showed favorable results of viability and induced a low-level inflammatory response when compared with NaOCl.^8^

Nevertheless, aqueous solutions of NaOCl and Ca(OCl)_2_ have high surface tension values.^3^ ^,^ ^7^ ^,^ ^9^ Surface tension is related to the wetting ability, surface free energy or capillarity effects.^10^ ^-^ ^12^ It allows solution penetration into both the main and lateral canals and into the dentinal tubules.^1^ ^,^ ^12^ On the other hand, surface free energy is the result of intermolecular interactions between the surface and the liquid. It can be expressed as the contact angle between a drop of liquid on a solid surface.^10^ ^,^ ^11^ The lower the surface tension and contact angle, the better the wettability potential of the solution.^11^

Cetrimide (Cet) is a cationic surfactant (quaternary ammonium salt) available as a white odorless powder and, according to the manufacturer, it is highly hygroscopic. It has the capacity of lowering surface tension,^12^ and antimicrobial activity.^13^ ^-^ ^15^ Benzalkonium chloride (BAK) is also a cationic surfactant, already used in dental clinical practice and associated with some endodontic irrigants.^16^ The antibacterial potential of BAK is associated with changes promoted on the ionic resistance of the cell membrane.^17^

The addition of surfactants to NaOCl as an alternative to decrease their high surface tension has been reported in the past.^9^ ^,^ ^12^ ^,^ ^16^ ^,^ ^18^ Furthermore, previous studies have shown bactericidal activity even when surfactants were used^19^ ^,^ ^20^. However, there are no data concerning the effect of the addition of surfactants to Ca(OCl)_2_.

Therefore, the objective of this study was to add surfactants Ca(OCl)_2_ and evaluate the effect on the properties of pH, chlorine content, surface tension, contact angle, pulp dissolution and antimicrobial capacity.

## Material and methods

This study was approved by the Institutional Review Board (Protocols n. 29495 and 59004516.4.0000.5347).

### Preparation of NaOCl and Ca(OCl)_2_ solutions

The solutions were titrated by the sodium thiosulfate method as described by Vogel^21^ (1965), and prepared immediately before the experiments. An aliquote of 10 mL of the tested solution was added to 90 mL of distilled water. 15 mL of the diluted solution was transferred to a 250 mL Erlenmeyer flask. To stain the sample, 1 mL of the potassium iodide solution and 1.7 mL of the 0.1 N sulfuric acid solution were added. Titration was performed with 0.1 N sodium thiosulfate until the solution in question became clear. Chlorine content was calculed as follows:

Calculus 1:

0.0036 g chlorine – 1 mL sodium thiosulfate

a - thiosulfate used in titratrion (mL)

Calculus 2:

15 mL – a (chlorine in 15mL)

100 mL – b (active chlorine in the diluted solution)

Calculus 3:

b × 10 (solution was diluted ten times)

A 12% NaOCl solution (Farmaquímica S.A. Produtos Químicos; Porto Alegre, Rio Grande do Sul, Brazil) was diluted in sterilized and distilled water to reach the 2.5% concentration. To obtain a 2.5% Ca(OCl)_2_ solution, 3.8461 g of Ca(OCl)_2_ powder, with 65% purity (Farmaquímica S.A. Produtos Químicos; Porto Alegre, Rio Grande do Sul, Brazil), was mixed with 100 mL of sterile distilled water. All solutions were prepared under constant agitation and were stored in bottles identified with random numbers for blinding.

The surfactants added to Ca(OCl)_2_ and NaOCl solutions were 0.2% cetrimide (0.2% Cet)^13^ ^-^ ^15^, 0.1% cetrimide (0.1% Cet)^19^ or 0.008% benzalkonium chloride (BAK).^16^ Distilled water (DW) was used as control group, as follows:

Group I: control, distilled water

Group II: 2.5% NaOCl

Group III: 2.5% NaOCl + 0.2% cetrimide

Group IV: 2.5% NaOCl + 0.1% cetrimide

Group V: 2.5% NaOCl + 0.008% BAK

Group VI: 2.5% Ca(OCl)_2_

Group VII: 2.5% Ca(OCl)_2_ + 0.2% cetrimide

Group VIII: 2.5% Ca(OCl)_2_ + 0.1% cetrimide

Group IX: 2.5% Ca(OCl)_2_ + 0.008% BAK

Group X: 0.2% cetrimide

Group XI: 0.1% cetrimide

Group XII: 0.008% BAK

### pH measurement

The pH (n=3 for each solution) was obtained immediately after manipulation and triplicate. Each sample was measured by a digital pHmeter (Digimed; São Paulo, São Paulo, Brazil). Calibration was performed according to the manufacturer's instructions, and a buffer at a pH of 10 was used as a control for each analysis.

### Determination of the available chlorine content

The available chlorine content (n=3 for each solution) was evaluated by sodium thiosulfate titration^21^ as described previously, and triplicate. The result was expressed in grams of active chlorine *per* 100 mL solution.

### Surface tension measurement

Surface tension was evaluated by the “ring method”.^22^ The Du Noüy tensiometer (Sigma; Attension, Espoo, Finland) was used in a room at constant temperature (25°C).

The equipment measures the strength needed to separate the platinum ring from within a solution after it has been submerged. Distilled water was used as the control and to perform initial calibration of the instrument. Twenty mL of the tested solution was positioned so that the ring could submerge. As the equipment slowly removed the ring from the liquid, it recorded solutions surface tension value. The test was performed in 3 samples of each solution and triplicate. Results were expressed in mN/m.

### Contact angle measurement

Thirty single-rooted extracted human teeth were obtained. The crown was removed at the cementoenamel junction, and the roots were longitudinally sectioned in two halves, mesial and distal. Each half was transversely divided in 2 pieces, cervical and apical. Four dentin specimens were obtained from each tooth. A total of 120 specimens (10 *per* group) were obtained, and stratified so that each group received 5 cervical and 5 apical specimens.

The specimens were embedded in self-curing acrylic resin (Jet Clássico; Campo Limpo Paulista, São Paulo, Brazil) with the root canal surface facing up. The surfaces were polished under running water using 80-, 100-, 120-, 150- and 180-grit abrasive papers to obtain a flat wide dentin surface.^10^ ^,^ ^16^ ^,^ ^20^

Contact angle measurements were achieved at 22°C by using a drop shape analysis system DSA100 (Kruss; Hamburg, Hamburg, Germany). A drop with 2 µl volume of tested solution was carefully placed with a micropipette on each dentin surface. After 30 seconds, the image of the droplet was obtained and the contact angle was calculated.^10^ ^,^ ^16^ ^,^ ^20^

Three parallel measurements were performed with each tested solution on both cervical and apical thirds.

Dentin specimens were polished to create a smooth and flat dentin surface,^16^ otherwise the program would not be able to measure contact angle inside the concave surface of root canal walls. The samples did not receive any superficial treatment, in order to simulate the presence of smear layer during root canal preparation and to observe solutions ability to penetrate in dentine tubules.

### Pulp dissolution test

Bovine pulp tissue was employed for the experiment. Teeth were extracted and stored at −20°C until required. The crowns were removed at the cementoenamel junction and the pulp tissue was removed. One-hundred twenty standardized pulp fragments (10 *per* group) with length of 5 mm and weight ranging from 0.015 and 0.025 g were prepared using a scalpel blade number 15 (MedGolman; São José, Santa Catarina, Brazil). The initial weight of each specimen was measured with a precision scale M1203 (BEL Engeneering; Monza, Monza and Brianza, Itália) in an airtight container. After weight recording, the specimens were randomly divided.

The pulp fragment was placed in a cell culture well and each specimen was irrigated with a total volume of 10 mL of solution, for 10 minutes. One mL of the solution was replaced every minute. At the end of the irrigation time, the specimens were removed from the wells and left in contact with a sterile absorbent paper for 30 seconds to remove the excess solution. The specimens were weighed to assess their final weight. The percentage of weight loss was determined for each sample.^23^ ^-^ ^25^ All specimens were weighed by a single investigator, which was unaware of how each was to be treated.

### Antimicrobial test

The agar diffusion method was used to measure the antimicrobial activity. The test was performed in 3 samples of each solution and triplicate. The strain used in the analysis was *Enterococcus faecalis* (ATCC 29212). The subcultures were incubated at 37°C for 48 hours prior to testing of the solutions and their base components. The purity of strains was checked by subculture of the inocula onto Brain Heart Infusion Agar (Himedia Laboratories Limited; Ghatkopar West, Mumbai, India) supplemented with 5% defibrinated sheep blood for *E. faecalis.* Agar well diffusion assays were performed in 90-mm diameter Petri dishes containing Mueller Hinton Agar (Himedia Laboratories Limited; Ghatkopar West, Mumbai, India) for *E. faecalis* at a depth of 4 mm. A direct colony suspension isolate was prepared in 0.85% sterile saline, and the turbidity was adjusted to a 0.5 McFarland standard. The agar plates were flooded with the test suspension and sterilized filter paper disks containing 20 µL of test solution were placed over the agar. The plates were then incubated at 37°C for 24 hours. After incubation, the diameters of the growth inhibition zones were measured in millimeters to the nearest 0.1 mm using electronic calipers (Digimess Instrumentos de Precisão Ltda; São Paulo, São Paulo, Brazil).

### Statistical analysis

The data were submitted to a normality test (Kolmogorov-Smirnov) and homocedasticity test (Levene). For free chlorine, surface tension, pulp dissolution and antimicrobial activity tests, since data were normal and had homocedasticity, One-way ANOVA was used, followed by Tukey *post hoc*. For contact angle tests, Kruskal-Wallis followed by paired comparison was used. Data analysis was performed by SPSS (Statistical Package for Social Science) for Mac version 20 at a 5% significance level.

## Results

### pH measurement

All NaOCl and Ca(OCl)_2_ with or without the addition of surfactants had a pH level above 11. Surfactants alone and DW had pH values around 7 (Table 1).

**Table 1:** pH readings of tested solutions (median, maximum and minimum values)

Group	pH values (max/min)
DW	7.76 (7.66-8.18)
NaOCl	12.88 (12.86-12.94)
NaOCl + 0.2% Cet	12.72 (12.68-12.76)
NaOCl + 0.1% Cet	12.76 (12.74-12.77)
NaOCl + BAK	11.80 (11.78-11.89)
Ca(OCl)^2^	12.65 (12.59-12.66)
Ca(OCl)_2_ + 0.2% Cet	11.89 (11.88-11.96)
Ca(OCl)_2_ + 0.1% Cet	11.87 (11.84-11.87)
Ca(OCl)_2_ + BAK	11.77 (11.56-11.80)
0.2% Cet	7.10 (6.82-7.35)
0.1% Cet	6.35 (6.29-6.50)
BAK	7.03 (6.75-7.15)

### Determination of the available chlorine content

DW, 0.2% and 0.1% Cet, and BAK did not present chlorine content and were not included in the statistical analysis. NaOCl + 0.1% Cet and NaOCl + BAK had the higher amount of chlorine content, with a statistical difference to Ca(OCl)_2_ solutions, with or without surfactant addition (*p*<0.05) (Table 2).

**Table 2:** Avaliable chlorine content (mean±SD)

Group	Chlorine content
NaOCl	2.93±0.13^abc^
NaOCl + 0.2% Cet	3.01±0.16^ab^
NaOCl + 0.1% Cet	3.16±0.19^a^
NaOCl + BAK	3.24±0.07^a^
Ca(OCl)_2_	2.73±0.08^bc^
Ca(OCl)_2_ + 0.2% Cet	2.77±0.08^bc^
Ca(OCl)_2_ + 0.1% Cet	2.58±0.09^c^
Ca(OCl)_2_ + BAK	2.63±0.08^c^

Different letters indicate statistical difference between solutions

ANOVA (Tukey *post hoc*) (p<0.05)

### Surface tension measurement

Ca(OCl)_2_ had the highest surface tension among all tested solutions. Surfactant addition to NaOCl and Ca(OCl)_2_ lowered the surface tension values, independently of the surfactant added, with statistical difference (*p*<0.05) (Table 3).

**Table 3:** Surface tension measurement in mN/m. (mean±SD)

Group	Surface tension
DW	52.07±1.13^b^
NaOCl	46.30±3.94^c^
NaOCl + 0.2% Cet	33.08±0.07^ef^
NaOCl + 0.1% Cet	32.85±0.22^ef^
NaOCl + BAK	30.92±0.24^f^
Ca(OCl)_2_	72.13±1.82^a^
Ca(OCl)_2_ + 0.2% Cet	32.89±0.93^ef^
Ca(OCl)_2_ + 0.1% Cet	33.67±0.85^ef^
Ca(OCl)_2_ + BAK	31.86±0.18^f^
0.2% Cet	36.99±0.28^de^
0.1% Cet	40.85±0.73^d^
BAK	50.53±2.20^bc^

Different letters indicate statistical difference between solutions

ANOVA (Tukey *post hoc*) (p<0.05)

### Contact angle measurement

**Figure 1:**
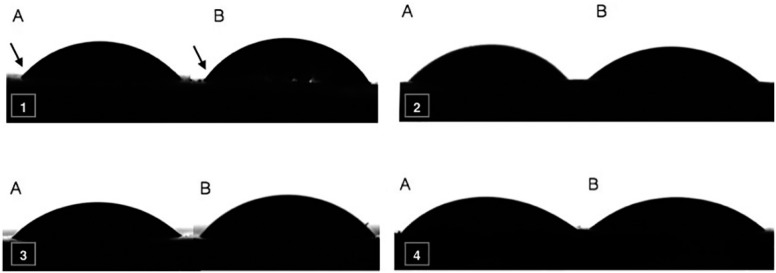
Representative images of solutions contact angle analysis on dentin surfaces (1) DW; (2) NaOCl; (3) Ca(OCl)_2_; (4) BAK. (A) cervical third; (B) apical third

Representative images of the analyzed groups (DW, NaOCl, Ca(OCl)_2_ and BAK) can be found in Figure 1.

Contact angle values <10° are not measured by the system. NaOCl and Ca(OCl)_2_ with the addition of surfactants, and 0.2% and 0.1% Cet alone had a contact angle <10°, for both cervical and apical specimens, and were not included in statistical analysis.

There was no statistical difference among cervical and apical specimens when the same solution was evaluated (*p*>0.05). Statistical difference was observed between Ca(OCl)_2_ and BAK, in both cervical (*p*=0.042) and apical (*p*=0.041) thirds. Ca(OCl)_2_ presented higher contact angle than BAK (Table 4).

**Table 4:** Contact angle measurement in cervical and apical thirds (median, maximum and minimum values)

Group	Cervical third	Apical third
DW	48.10 (38.14-55.31)^ab^	50.58 (49.95-61.07)^AB^
NaOCl	44.27 (29.16-51.97)^ab^	44.63 (40.51-50.57)^AB^
Ca(OCl)_2_	53.17 (43.54-61.82)^ab^	52.85 (48.46-64.65)^A^
BAK	42.58 (28.01-45.32)^b^	42.84 (29.29-47.00)^B^

Different letters indicate statistical difference between solutions in the same third. Kruskal-Wallis (paired comparison) (p<0.05)

### Pulp dissolution test

NaOCl solutions, with and without surfactant addition, dissolved more pulpal tissue than all other tested solutions. Ca(OCl)_2_ solutions presented pulpal dissolution ability. Surfactants alone did not have dissolution ability, as well as DW (Table 5).

**Table 5:** Pulp dissolution test (mean percentage reduction in weight of specimens) (mean±SD)

Group	Pulp dissolution
DW	-40.67 ± 25.24^d^
NaOCl	88.13±11.39^a^
NaOCl + 0.2% Cet	83.62±9.83^a^
NaOCl + 0.1% Cet	84.39±4.73^a^
NaOCl + BAK	86.47±6.40^a^
Ca(OCl)_2_	39.53±7.23^b^
Ca(OCl)_2_ + 0.2% Cet	40.03±7.23^b^
Ca(OCl)_2_ + 0.1% Cet	40.41±15.54^b^
Ca(OCl)_2_ + BAK	38.52±9.72^b^
0.2% Cet	-4.42 ± 5.02^c^
0.1% Cet	-12.97 ± 12.27^c^
BAK	-50.65 ± 20.42^d^

Different letters indicate statistical difference between solutions

ANOVA (Tukey *post hoc*) (p<0.05)

### Antimicrobial test

Ampicillin (positive control) had the highest antimicrobial inhibition zone. All solutions, except DW, had antimicrobial activity against *E. faecalis*. Addition of 0.2 or 0.1% Cet to NaOCl and Ca(OCl)_2_ enhanced their inhibition zone (*p*<0.05) (Table 6).

**Table 6:** Inhibition zone against *Enterococcus faecalis* (mm) (mean±SD). Ampicilline was the positive control

Group	Inhibition zone
DW	0.00^g^
NaOCl	13.76±2.06^ef^
NaOCl + 0.2% Cet	21.21±1.35^b^
NaOCl + 0.1% Cet	19.77±4.55^bc^
NaOCl + BAK	16.69±2.07^cde^
Ca(OCl)_2_	14.23±1.65^ef^
Ca(OCl)_2_ + 0.2% Cet	20.84±1.01^b^
Ca(OCl)_2_ + 0.1% Cet	18.40±2.12^bcd^
Ca(OCl)_2_ + BAK	16.72±2.79^cde^
0.2% Cet	18.50±1.08^bcd^
0.1% Cet	16.52±0.98d^e^
BAK	12.18±2.07^f^
Ampiciline	31.60±0.86^a^

Different letters indicate statistical difference between solutions

ANOVA (Tukey *post hoc*) (p<0.05)

## Discussion

Root canal irrigation plays a fundamental role in clinical endodontic success. The main irrigant should be able to spread along all root canal structures, enabling the clinician to promote better cleaning and shaping. The addition of surfactants to the Ca(OCl)_2_ solution reduced its surface tension, possibly enhancing its wetting ability, which may enable a better diffusion of the irrigant on dentin walls,^16^ leading to improved action of the solution during endodontic treatment.

A digital pHmeter was used to determine the pH of solutions, as already described by other studies.^3^ ^,^ ^12^ ^,^ ^15^ ^,^ ^26^ ^-^ ^28^ Ca(OCl)_2_ and NaOCl are strongly alkaline solutions.^3^ ^,^ ^15^ ^,^ ^18^ ^,^ ^26^ Even though the surfactants showed a lower pH than Ca(OCl)_2_ and NaOCl, their addition to the solutions did not alter their pH, agreeing with other study.^18^ This could be related to the lower concentration of surfactants compared to Ca(OCl)_2_ and NaOCl (two and three orders of magnitude lower for cetrimide and BAK, respectively).

Since surfactants did not alter the pH of solutions, they may not affect the properties directly related to it, as antimicrobial activity and pulp dissolution. Additionally, the non-polar long tail group of the surfactants and the low concentration used in the present work allow dissolution into a low surface free energy interface, such as the pulp. At lower pH, available chlorine is in the form of hypochlorous acid,^1^ which has greater antimicrobial activity and is more cytotoxic.^26^ Moreover, solution becomes more unstable, losing a significant amount of free chlorine content and, consequently, its action potential.^29^ On the other hand, at higher pH values, available chlorine is in the form of hypochlorite ions,^1^ which has less antimicrobial activity but is also less cytotoxic.^29^

The hypochlorite concentration can be affected by many factors, such as temperature and storage conditions,^3^ ^,^ ^15^ and for this reason it is important to know quantitatively the free chlorine content before use. In the present work the standard thiosulphate titration method was used to obtain accuracy in actual hypochlorite concentration.^3^ ^,^ ^29^

The titration results showed that surfactant addition did not alter Ca(OCl)_2_ and NaOCl free chlorine content, as already described for NaOCl solutions.^16^ ^,^ ^18^ ^,^ ^27^ This could be explained by the low concentration of surfactants used in this study, which would not be able to affect chlorine concentration of Ca(OCl)_2_ and NaOCl. In addition, the titration results showed that all hypochlorite solutions prepared had a higher amount of chlorine than 2.5%, demonstrating the importance of the thiosulphate titration method to know the actual hypochlorite concentration. Also, analysis of the free chlorine content of solutions allows knowing their ability to act against microorganisms and organic tissue.

Facultative bacteria, such as *Enterococus faecalis* have been considered one of the most critical endodontic pathogens in endodontic infections, and its presence can lead to root canal treatment failure.^30^ Although Ca(OCl)_2_ has more hypochlorous acid release,^5^ there was no difference in antimicrobial activity when compared to NaOCl, agreeing with other authors.^7^ The theoretical concentrations of free OCl^-^ used in the present study for Ca(OCl)_2_ and NaOCl were 0.17 mol/L and 0.33 mol/L respectively, which indicate that both hypochlorite solutions release approximately equal amounts of free chlorine and therefore produced similar antimicrobial activities. The surfactants used in this study have antimicrobial activity, agreeing with the results from previous studies.^13^ ^-^ ^15^ Their molecules are positively charged and bind strongly to bacterial cell walls and membranes because of their opposite negative charge, leading to progressive leakage of cytoplasmatic materials, causing cell disruption.^31^

The antimicrobial activity was improved compared with pure hypochlorite solutions when Cet was added at concentrations of 0.2% and 0.1%. The addition of BAK did not enhance the inhibition zone of NaOCl and Ca(OCl)_2_ solutions, agreeing with Bukiet, et al.^16^ (2012). This can be explained by the lower concentration of BAK when compared to Cet. The BAK concentration was approximately 17 and 8 times lower than the Cet concentrations (0.2% and 0.1%, respectively). However, the 0.008% BAK concentration was previously tested with NaOCl solution, and besides lowering surface tension values, it did not affect its free chlorine content, cytotoxicity, and antimicrobial properties.^16^ The antimicrobial activity of Ca(OCl)_2_ and NaOCl solutions was similar, with no statistical difference between them.

The agar diffusion method has some limitations, such as substance ability to spread through agar, number of microorganisms inoculated, substrate pH value, agar viscosity, storage conditions, incubation period and microorganisms metabolic activity.^29^ Moreover, it is important to consider that the solution was in direct contact with the microorganisms, and clinically this does not occur throughout the entire root canal system, due to anatomical structures.^29^ However, this is one of the most common tests used for evaluating antibacterial activity against a chemical agent, and, also, it can be considered a relevant protocol.^32^

Dissolution has been studied in bovine pulp tissue,^2^ human pulp tissue,^33^ bovine muscle tissue.^5^ ^,^ ^20^ Concerning the irrigant, few studies evaluated the tissue dissolution ability of Ca(OCl)_2_.^5^ ^,^ ^6^ None of the tested surfactants had pulp dissolving ability, which can be explained by their absence of free chlorine content. Therefore, surfactant addition did not alter the dissolution ability, agreeing with other authors, who tested NaOCl at different concentrations.^24^ ^,^ ^27^ ^,^ ^33^ However, other authors found that surfactant addition resulted in better NaOCl dissolution ability.^20^ ^,^ ^27^

The results showed that Ca(OCl)_2_ presented lower dissolution ability than NaOCl. One of the concerns related to NaOCl dissolution ability is the harm to periapical tissues in case of accidental outflow.^1^ It has been suggested that Ca(OCl)_2_ could be less aggressive to periapical tissues.^5^ In this regard, Ca(OCl)_2_ could have favorable results of viability and induced low-level inflammatory response, mainly in infected immature teeth treatment.

The present study observed that specimens immersed in Ca(OCl)_2_ developed a white surface coating on the entire pulp fragment, as already described.^3^ ^-^ ^5^ According to Dutta, et al.^5^ (2012), this coating can be calcium hydroxide that may have bonded to the tissue as part of amino acid reactions of saponification and neutralization.

Leonardo, et al.^3^ (2016) observed the formation of a white precipitate in the bottom of plastic tubes used for Ca(OCl)_2_ solution storage. Its composition was measured with energy-dispersive X-ray spectroscopy, which enabled detection of the individual elements of particles that were formed predominantly by calcium. The average pH measured for the Ca(OCl)_2_ solutions was about 12.6, which means a OH^-^_(aq)_ equilibrium concentration of about 0.04 mol/L. Taking into account that the solubility product constant at 25°C for Ca(OH)_2_ is 5.5 × 10^-6^,^34^ a simple calculation of the OH^-^_(aq)_ equilibrium concentration reveals a value of 0.022 mol/L. Because the actual OH^-^_(aq)_ is higher than the equilibrium concentration, Ca(OH)_2_ will precipitate at 25°C. The formation of calcium hydroxide covering the pulp tissue could affect the action of chlorine, lowering its dissolution effect.

Surface tension measurement by the Du Noüy tensiometer was already described by other authors^3^ ^,^ ^22^. The value is calculated by the equipment and was expressed in mN/m, but it can also be expressed in other measurement unit such as dyne/cm or mJ/m^2^. Since the ring method does not consider the dentin surface,^16^ it is important to analyze not only the surface tension but also the contact angle. Surface tension reduction and lower contact angle values can improve the contact between irrigant and the dentin walls.^9^ ^,^ ^12^

If the contact angle value is lower than 90°, the substrate is wetted by the liquid; if the contact angle is greater than 90°, the liquid is considered non-wetting. A contact angle value equal to zero represents complete wetting.^11^ Thus, the lower the contact angle, the faster the liquid will spread through the dental surface.^10^

The addition of surfactants in both Ca(OCl)_2_ and NaOCl resulted in complete wetting of the dentin specimens. Among surfactants, only BAK solution had a contact angle measured by the software, yet this did not occur when it was added to hypochlorite. However, in the clinical reality, factors such as surface irregularities and natural moisture of the dentin, will influence this behavior.

This is the first study to evaluate surfactant addition to Ca(OCl)_2_ solutions. The prepared Ca(OCl)_2_ solutions had the higher surface tension measured among all solutions herein tested (72.13±1.82 mN/m), agreeing with Leonardo, et al.^3^ (2016). When surfactants were added to the Ca(OCl)_2_ solutions, there was a decrease in the surface tension of all prepared solutions, reaching similar values to the NaOCl solutions. The lowering of surface tensions when the surfactants were added to the hypochlorite solutions explains the decrease in the contact angle measured, reaching complete wetting of the dentin specimens.

Even with the limitations of the *in vitro* tests, this study observed that surfactant addition to Ca(OCl)_2_ has shown acceptable outcomes for pH, free chlorine content, surface tension, contact angle, pulp dissolution and antimicrobial activity and physicochemical properties, which may qualify it as an irrigant in endodontic practice. When compared to NaOCl, the standard irrigant solution, Ca(OCl)_2_ appears to be less cytotoxic^8^ and more stable.^3^ Also, the addition of 0.2% Cet showed better results regarding the antimicrobial activity, contact angle and surface tension values. The possibility of mixing the Ca(OCl)_2_ solution right before its use could provide more certainty to the dentist of having a more reliable, stable solution for clinical use. Further studies should be performed to evaluate Ca(OCl)_2_ with addition of surfactants as root canal irrigant in the clinical practice.

## Conclusion

Surfactant addition to Ca(OCl)_2_ has shown acceptable outcomes for pH, free chlorine content, surface tension, contact angle, pulp dissolution and antimicrobial activity, physicochemical properties that can qualify it as an irrigant in Endodontics. Furthermore, the addition of 0.2% Cet showed better results for all tested properties.
